# Music Improvisation Is Characterized by Increase EEG Spectral Power in Prefrontal and Perceptual Motor Cortical Sources and Can be Reliably Classified From Non-improvisatory Performance

**DOI:** 10.3389/fnhum.2019.00435

**Published:** 2019-12-10

**Authors:** Masaru Sasaki, John Iversen, Daniel E. Callan

**Affiliations:** ^1^Graduate School of Frontier Biosciences, Osaka University, Osaka, Japan; ^2^Swartz Center for Computational Neuroscience, University of California, San Diego, San Diego, CA, United States; ^3^Center for Information and Neural Networks (CiNet), National Institute of Information and Communications Technology (NICT), Osaka University, Osaka, Japan

**Keywords:** music, improvisation, machine learning, loreta, ICA, EEG, guitar, medial frontal cortex

## Abstract

This study expores neural activity underlying creative processes through the investigation of music improvisation. Fourteen guitar players with a high level of improvisation skill participated in this experiment. The experimental task involved playing 32-s alternating blocks of improvisation and scales on guitar. electroencephalography (EEG) data was measured continuously throughout the experiment. In order to remove potential artifacts and extract brain-related activity the following signal processing techniques were employed: bandpass filtering, Artifact Subspace Reconstruction, and Independent Component Analysis (ICA). For each participant, artifact related independent components (ICs) were removed from the EEG data and only ICs found to be from brain activity were retained. Source localization using this brain-related activity was carried out using sLORETA. Greater activity for improvisation over scale was found in multiple frequency bands (theta, alpha, and beta) localized primarily in the medial frontal cortex (MFC), Middle frontal gyrus (MFG), anterior cingulate, polar medial prefrontal cortex (MPFC), premotor cortex (PMC), pre and postcentral gyrus (PreCG and PostCG), superior temporal gyrus (STG), inferior parietal lobule (IPL), and the temporal-parietal junction. Together this collection of brain regions suggests that improvisation was mediated by processes involved in coordinating planned sequences of movement that are modulated in response to ongoing environmental context through monitoring and feedback of sensory states in relation to internal plans and goals. Machine-learning using Common Spatial Patterns (CSP) for EEG feature extraction attained a mean of over 75% classification performance for improvisation vs. scale conditions across participants. These machine-learning results are a step towards the development of a brain-computer interface that could be used for neurofeedback training to improve creativity.

## Introduction

The neural processes underlying creativity is a topic of great interest. There has been considerable research that has yielded valuable insights into possible neural correlates involved in creative thinking (Mölle et al., 1999; Jung-Beeman et al., 2004; Fink and Neubauer, 2006). However, experimental tasks that are used in this field of research are often very simple and far removed from real-life experience. They may be indicative only of basic aspects of creative thinking. Research in this field also needs to consider the neuroergonomic investigation of more complex, “real-life” tasks involving creative processing. Music improvisation is an example of such a complex, “real-life” creative activity that we will investigate in this study. Improvisation is the process of generating something novel and esthetically appealing, spontaneously in real-time, while evaluating and coordinating ongoing performance in relation to relevant context (Beaty, 2015).

Functional magnetic resonance imaging (fMRI) studies have identified a myriad of brain regions active during music improvisation relative to control conditions. The general processing characteristics of these regions has given some insight into their collective role in music improvisation. A review by Beaty (2015) puts forward that music improvisation involves task and context-dependent interaction between brain networks involved with internally directed value mediated attention/spontaneous cognition [polar medial prefrontal cortex (MPFC), medial frontal cortex (MFC)] and those involved with externally directed attention/cognitive control [inferior frontal gyrus (IFG), premotor cortex (PMC), and dorsolateral prefrontal cortex (DLPFC)]. Several studies have reported improvisation related activity in the superior temporal gyrus (STG), inferior parietal lobule (IPL), temporal-parietal junction Temporal parietal junction (TPJ), and PMC (Beaty, 2015). These regions are involved in part with feedback regulation of perceptual-motor processing and control that is an integral component of music improvisation.

Consistent with many fMRI studies, electroencephalography (EEG) and MEG research has found improvisation related activity in MFC and DLPFC regions in the alpha (8–12 Hz) and Beta (13–30 Hz) frequency range (Dolan et al., 2013; Boasen et al., 2018; Stevens and Zabelina, 2019). Although there are a large number of studies investigating music improvisation using EEG many of them only employ sensor level analyses, making it difficult to make mechanistic inferences (Müller et al., 2013; Wan et al., 2014; Dikaya and Skirtach, 2015; Sanyal et al., 2016; Lopata et al., 2017; Dolan et al., 2018).

It is our goal to extend past EEG studies of music improvisation by using advanced methods that permit analysis at the cortical source level: high-density EEG recording (64 channel), cortical source localization, advanced artifact extraction, and machine learning techniques. Unlike fMRI, where there are considerable constraints in the body position and possible hand and arm motion made by the participant, EEG allows for experiments using natural body posture and movement. EEG may have advantages in conducting research related to performing music in this respect as well as the lack of the presence of loud acoustic noise created by fMRI scanning.

Using an experimental task similar to that implemented by Limb and Braun (2008) and Berkowitz and Ansari (2008), guitar musicians performed alternating blocks of improvisation and scales within a key defined by a tonic context chord played continuously in the background (see “Materials and Methods” section). This background context is important for setting up melodic constraints (Limb and Braun, 2008) and rhythmic constraints (Berkowitz and Ansari, 2008) for improvisation. Because of possible artifacts associated with hand, arm, and body movement while playing music, advanced artifact correction and extraction techniques are used including artifact subspace reconstruction ASR (Mullen et al., 2013; Chang et al., 2018) and independent component analysis (ICA; Delorme and Makeig, 2004) to separate artifact and brain activity. Research has shown that ICA can be used to extract brain-related activity even in challenging recording environments during walking and/or moving (Artoni et al., 2017; Wagner et al., 2019) as well as during piloting of airplanes (Callan et al., 2015, 2018). Previous EEG research concerned with music improvisation has not used techniques such as ICA to separate brain activity from physiological and movement-based artifacts. Most EEG music improvisation studies use only simple band-pass filtering and amplitude-based trial rejection as well as subjective visual inspection techniques (Dolan et al., 2013, 2018; Müller et al., 2013; Wan et al., 2014; Dikaya and Skirtach, 2015; Adhikari et al., 2016; Sanyal et al., 2016; Lopata et al., 2017).

Based on previous fMRI and EEG/MEG research it is predicted that improvisation over scale will be characterized by increased alpha and beta frequency range power in brain regions involved with internally mediated cognitive processing (MFC, polar MPFC), externally directed cognitive control (DLPFC, IFG), and feedback regulated perceptual-motor planning/control (STG, IPL, TPJ, PMC). An additional goal of our research is to determine if individual-specific brain-related activity could be used to classify whether a musician is playing improvisation or non-improvised scales using machine-learning techniques, as a prelude to developing a neurofeedback device capable of detecting and rewarding improvisatory states.

## Materials and Methods

### Participants

The participants in this study consisted of 14 male adults aged 20–41 years (mean = 25.12, SE = 1.12) who were proficient at playing the guitar. Thirteen of the participants were right-handed and one participant was left-handed. The hand used to write and use tools determined handedness. All participants play right-handed guitar.

The Osaka University experiment recruitment Twitter account was used to find potential participants for the experiment. The participants included in the study were selected based on their musical experience as reported by questionnaire. The most important questions for inclusion were experience and style of improvisation as well as experienced in the composition of songs. All participants included had at least 2 years of experience with improvisation using the guitar and 1 year of experience composing songs. Originally there were 18 participants recruited but four participants were eliminated from the study for various reasons including extremely noisy EEG data (two participants) and improperly performing the experimental task (two participants). The experimental procedures were approved by the NICT Human Subject Review Committee and were carried out in accordance with the principles expressed in the WMA Declaration of Helsinki.

### Experimental Task and Procedure

In this experiment, we explored brain activity associated with music improvisation using the guitar. The guitar was selected over other musical instruments for two primary reasons: (1) the extensive guitar playing experience of the first author of this study, and (2) ease of recruiting participants with improvisation guitar experience. The guitar used in this study was the ST62US made by Fender Japan. The experiment was conducted in a sound-attenuated room at the Center for Information and Neural Networks.

The experimental task used to probe brain activity underlying musical improvisation was based in part on methods used in the study by Berkowitz and Ansari (2008) and Limb and Braun (2008). To account for perceptual and motor components of playing the guitar that is not uniquely related to improvisation a control condition was employed in which an ascending major scale (one octave) was played in accompaniment with audio presentation (by computer speaker; Alienware 15R3 Laptop PC using windows 10) of a specific chord. There were three chords in total: A major, Bb major, and Eb major. The order of the chord presented was random. This chord served as a background music context. Throughout the experiment, a metronome (iPhone application: practice+ made by Dynamic App Design LLC) was playing at a speed of 80 BPM (beats-per-minute) of four beat measures. This control condition we called “Scale.” The notes of the Scale condition were performed as eighth notes. For the experimental “Improv” condition participants were asked to generate unique music in accompaniment with one of the three specific chords (A major, Bb major, and Eb major; randomly presented) with the restriction that the notes used were only from the specific major scale component of the currently presented chord. The tempo and rhythm of the Improv condition were flexible allowing for the use of quarter, eighth, eighth note triplets and 16th notes. The use of the metronome to control the pace as well as the restrictions of the notes played in the Improv condition were used to better control for motor and perceptual components that may differ between conditions and participants if left unconstrained.

**Figure 1:**
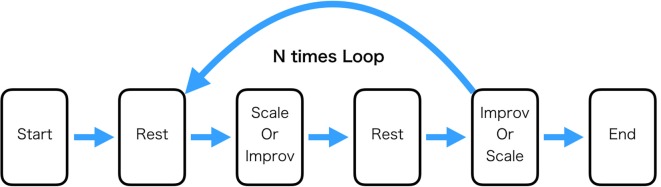
Experimental task procedure. The “Rest” condition consisted of the participant just sitting still. The “Scale” condition consisted of playing the major scale ascending to one octave higher. The “Improv” condition consisted of playing improvisation in a specified key that was the same as the “Scale” condition. One loop through the experiment consisted of playing Scale and Improv the order of which was randomized. The experiment consisted of 21 loops. In this experiment, three keys were used (Bb, Eb, and A). Each key was played seven times for a total of 21 loops.

The Scale and Improv conditions were 32 s each and were separated by 16 s of rest (participants were asked to do nothing during this period). The specific condition was signified by the word “Scale,” “Improv,” or “Rest” presented on the computer screen along with the audio presentation of the specific chord (A major, Bb major, or Eb major) the participant was to accompany playing the guitar. Matlab running on Alienware 15R3 Laptop computer was used to present the stimuli and send triggers of their onset to the EEG device. For one block consisting of Scale(Improv)-Rest- Improv(Scale)-Rest the same chord was used (Figure 1).

The order of the conditions in the block was constant across the experiment for a single participant. The order of whether Scale or Improv occurred first was counterbalanced across participants and randomly determined. It was believed that randomizing the sequence order within a single participant would cause too many errors of playing the appropriate condition. Each of the three chords was presented seven times for a total of 21 occurrences of each condition (Scale and Improv) throughout the experiment (approximately 35 min). The participants were introduced to the three context chords prior to beginning the experiment. Audio and video of the guitar performance of each participant during the experiment was recorded using a GoPro Hero 6 camera.

### EEG Recording and Analysis

EEG was measured using the Cognionics HD-72 dry-wireless 64 channel EEG system (Cognionics Inc., San Diego, CA, USA). The sampling rate was 500 Hz with 24-bit analog to digital conversion. The system utilizes active electrodes as well as having active shielding covering all of the electrodes in the headset that are used to minimize external noise pickup and artifacts. See Callan et al. (2015, 2018) and Mullen et al. (2013) for more technical details regarding the Cognionics HD-72 EEG system used in this experiment. The data were wirelessly recorded using Cognionics software running on the experimental computer (Alienware 15R3). Triggers for the onset of the various conditions were sent wirelessly to the HD-72 and recorded on the EEG trace. The EEG was continuously recorded throughout the experiment.

The preprocessing steps given below were used to remove artifacts and improve signal quality to be able to extract task-relevant brain activity. Our overall analysis strategy used data cleaning and ICA to extract brain-related activity from the artifact, followed by cortical source analysis using LORETA to describe spatial patterns of activity in different frequency bands. Our machine learning strategy used frequency-band common spatial pattern analysis to drive a logistic regression classifier. An important aspect of our pipeline is that it is completely automated and requires no subjective evaluation. Analyses requiring subjective evaluation have a strong potential for experimenter bias, cannot be replicated, and do not lend themselves to neuroergonomic applications.

### Pre-processing and ICA

Processing of the EEG data was conducted using the EEGLAB toolbox (Delorme and Makeig, 2004) using a similar pipeline as given in Bigdely-Shamlo et al. (2015) and Callan et al. (2018), Pedroni et al. (2019), and those suggested on the EEGLAB wiki^1^.

- Analysis windows: for each experimental condition (improve and scale), the data from 4 to 28 s was retained. The first and last 4 s were left out to eliminate potential boundary effects of switching conditions. The 16 s rest condition between experimental trials were also cut out of the data. It was necessary to cut the rest condition out of the analysis because many subjects moved their arms, hands, and heads a lot during the rest condition creating excessive artifacts. Additionally, there was no way to ensure that participants were actually doing nothing (resting) rather than mentally rehearsing playing the guitar during this period. These two reasons, unfortunately, precluded analysis of the experimental conditions (improv and scale) relative to rest.
- Two datasets were constructed with bandpass filtering of raw continuous data from 2 to 30 Hz and 1 to 100 Hz using a Hamming windowed Sinc FIR filter. The dataset filtered from 2 to 30 Hz was used for determining the parameters used for preprocessing as well as the ICA weights. Using the 2–30 Hz dataset resulted in an ICA decomposition revealing more effective brain sources. The learned ICA weights were then applied to the 1–100 Hz dataset, which was used for all subsequent analysis. The use of high pass filtering at 1–2 Hz has been found to work best for ICA (Winkler et al., 2015). ICLabel used to identify artifact and brain independent components (ICs) utilizes data filtered from 1 to 100 Hz. This was the primary reason for selecting this frequency band.
- Automatic channel rejection using the 2–30 Hz filtered dataset was based on the following: flat channel duration (>5 s and poor correlation to robust estimate based on other channels (0.8; default parameter values were used: functions for automatic channel rejection were from the Clean Continuous Data Using ASR plugin). The channels that were rejected from the 2 to 30 Hz dataset were also rejected from the other 1 to 100 Hz dataset. It is important to remove bad channels to improve automatic data cleaning methods such as artifact subspace reconstruction (see below) as well as for prevention of spreading of artifacts throughout all channels during average referencing (Bigdely-Shamlo et al., 2015). Supplementary Figure S1 provides a topographic plot of the good (retained) and bad (rejected) channels for each participant. An analysis of the number of bad channels from the participants reveals that anterior channels (mean = 1.18; SE = 0.34; out 14 channels total; first two EEG channel rows) frontal central channels (mean = 3.07; SE = 0.91; out of 18 channels total; EEG channel rows 3 and 4) had relatively few bad channels compared to central parietal channels (mean = 6.93; SE = 0.94; out of 18 channels; EEG channel rows 5 and 6) and parietal occipital channels (mean = 5.57; SE = 0.66; out of 14 channels; EEG channel rows 7 and 8). The anterior channels have significantly fewer bad channels (normalizing for number of total channels) than central parietal channels (paired *T* = −5.81; *p* < 0.05) and parietal occipital channels (paired *T* = −6.60; *p* < 0.05). The frontal central channels also have significantly fewer bad channels (normalizing for number of total channels) than central parietal channels (paired *T* = −5.90; *p* < 0.05) and parietal occipital channels (paired *T* = −7.06; *p* < 0.05).
- Artifact subspace reconstruction (ASR; see Mullen et al., 2013; Chang et al., 2018) is a component-based method to remove transient and large amplitude non-stationary artifacts that is online and real-time capable. ASR was used to remove non-stationary high-variance signals from the EEG (standard deviation cutoff for removal of bursts = 20; Windowed Criterion = 0.25). For the 2–30 Hz dataset time windows that were not repaired using the 0.25 Windowed Criterion by ASR were removed. This was to provide a clean dataset for ICA training. The percentage of samples removed by ASR using the above given windowed criterion was 2.23% out of 504,084 samples (SE = 1.05%; the percent for each of the participants are as follows: 0.22%, 0.41%, 1.21%, 1.11%, 0.15%, 2.77%, 0.32%, 1.88%, 6.67%, 1.11%, 13.99%, 0.51%, 0.71%, 0.12%). It should be noted that ASR allows for cleaning of data such that trials that may be normally discarded can now be used for further analysis. This is extremely important when one considers neuroergonomic applications that operate in real-time in which all trials must be used.
- Interpolating the rejected channels using a spherical spline method was conducted for both datasets after ASR cleaning. It is important to interpolate the missing bad channels that have been removed before common average referencing to remove bias that may arise from concentrated (non-diffuse) bad channels (e.g., bad channels all being on the right hemisphere). Interpolating before ICA is necessary because afterward, it is not possible to recover the EEG.icasphere for the rejected bad channels. The EEG.icasphere is necessary to project ICs back to the electrodes used for sLORETA analysis (see below).
- Common average referencing of channels was conducted on both datasets after interpolation of missing channels. The primary reason for common average referencing is for the use of ICLabel that takes this type of data as input to identify brain and artifact components (Pion-Tonachini et al., 2019).
- ICA is a signal processing technique that separates linearly mixed sources at the level of sensors (EEG electrodes in our case) into independent components (Bell and Sejnowski, 1995). Infomax ICA (Bell and Sejnowski, 1995; Delorme and Makeig, 2004) using PCA reduction was used on the common average referenced and preprocessed 2–30 Hz dataset. The number of rejected channels determined the rank reduction by PCA with one added rank reduction for the use of common average referencing (see Supplementary Figure S1 for the number of PCA reduction used for each participant). The data with the removed time windows that could not be repaired by ASR was used for ICA to help improve decomposition. The goal for using ICA was to extract artifact data from brain data that may be mixed in several components. The goal was not explicitly to find independent task-related brain components.
- The weights of the ICA from the dataset filtered from 2 to 30 Hz were then applied to the ASR results without the time windows removed (Windowed Criteria setting turned off) of the 1–100 Hz dataset, which was used for cortical effective source identification. This procedure allows for all trials to be analyzed, which is important when considering neuroergonomic applications.
- To identify ICs representing effective cortical activity, while rejecting muscle and other artifacts, the following steps were taken: ICLabel (version 1.1) was used to identify ICs that are brain-related vs. artifact related^2^. ICLabel is a tool that allows for automated expert classification of ICs into seven different categories: Brain, Muscle, Eye, Heart, Line Noise, Channel Noise, and Other (Pion-Tonachini et al., 2019). ICLabel uses an artificial neural network to learn this classification from training on over 6,000 EEG recordings subjected to ICA for which experts have labeled components. This number of different EEG recordings (from different individuals and different EEG systems) used for training the classifier, is far greater than other automated IC classification tools (Pion-Tonachini et al., 2019). ICLabel performs better or as good as other current automated IC classification tools with greater efficiency in processing time (10 times faster; Pion-Tonachini et al., 2019). ICLabel uses IC topo maps and power spectral density (PSD) to categorize each IC (Pion-Tonachini et al., 2019). The criteria of selecting “Brian” ICs in our study was based on the percentage of “Brain” categorization over 60% and the sum of the artifact categories (Muscle, Eye, Heart, Line Noise, Channel Noise) was under 20%. We opted to use a very strict criterion for selection of brain-related ICs at the expense of potentially selecting only a few ICs for some of the participants to avoid contamination of the results by artifacts that are likely to be numerous given the real-world nature of our task. A primary advantage of using ICLabel instead of subjective evaluation (prone to experimenter bias) to select brain-related ICs is that it is automated and replicable. Brain-related ICs were retained and all other ICs were removed and data re-projected to the EEG electrodes in preparation for source localization (see below). See Supplementary Figure S1 for the topo maps of the ICLabel selected brain-related ICs for each participant.

### Source Localization

Source localization of brain-related activity on the surface of the cortex was determined using LORETA (Low-Resolution brain Electromagnetic Tomography) Key software employing sLORETA (Fuchs et al., 2002; Pascual-Marqui, 2002; Jurcak et al., 2007). LORETA is a low-resolution source localization technique that computes the generated electric neural activity in voxels that segment the gray matter of the brain. Only the ICs determined to be brain components by ICLabel were used. All other components containing artifacts were removed from the EEG data. The Electrode position on the head was determined by reference to the 10-10 system within the LORETA Key software. It should be noted that because generic head models were used instead of individual-specific models based on MRI anatomical images in which the position of the electrodes in relation to the brain can be precisely determined the absolute accuracy of the sLORETA source localization is limited, although localization is accurate at a regional level of resolution.

Source localization was computed for 21 improv and 21 scale trials in total, in the following frequency bands (theta 6.5–8 Hz; alpha1 8.5–10 Hz; alpha2 10.5–12 Hz; beta1 12.5–18 Hz; beta2 18.5–21 Hz; beta3 21.5–30 Hz; gamma 30.5–50 Hz; full alpha 8.5–12 Hz; full beta 12.5–30 Hz). The frequency bands were selected in part because they were defaults given in the Loreta Key software that are consistent with those given in Rangaswamy et al. (2002), Kropotov (2016), and Newson and Thiagarajan (2019). Another practical reason for using multiple sub-bands within traditional frequency ranges was to increase the number of features for machine learning analysis (see below). For each participant the individual trials were submitted to a statistical non-parametric (SnPM) analysis for the contrast of improv vs. scale. The resultant sLORETA map for this contrast for each participant was used in a random-effects group-level analysis using SnPM analysis (5,000 randomizations) within the LORETA Key software. Corrected critical thresholds for multiple comparisons were used in accordance with SnPM (Nichols and Holmes, 2001).

We believe that there are advantages in the analysis of differences between improv and scale at the cortical source localized level (using sLORETA) over that of conducting the analysis over the separate brain-related ICs. In this research our goal was to use ICA to remove artifacts from the EEG while retaining brain-related activity. The brain-related components were determined by ICLabel and then all other components were removed from the EEG data. Although it is possible to do spectral analysis of the IC activations contrasting improv vs. scale conditions for a single participant it is often difficult to find comparable components that are the same across all participants. One limitation of the cluster analyses used to group similar ICs across participants is that they can give variable results depending on the number of clusters the algorithms are set to find. An advantage of utilizing all brain-related ICs for the EEG data is that their mixture may result in differences in cortical source localized activity that may not be found when considering the individual ICs separately.

### Machine Learning

Machine learning was carried out over brain-related EEG data using Common Spatial Patterns (CSP; Blankertz et al., 2008; MNE, open-source Python software) for feature extraction and logistic regression (Tomioka and Aihara, 2007; Tomioka et al., 2007; Blankertz et al., 2008) for the classifier distinguishing between Scale and Improv conditions. Logistic regression is a statistical model used to predict a binary dependent variable using weighted predictor variables (features) by means of the log-odds (for formal details see Sperandei, 2014). These feature extraction and classification methods were selected because the algorithms are commonly used for two-group classification. An additional reason for using logistic regression as a classifier is that the model weights for each feature can be easily assessed, enabling the evaluation of what features of brain activity are most predictive. This is not true of many machine learning classification methods such as support vector machines (Liu et al., 2012; Selim et al., 2018). It is important to note that the EEG data used for machine learning consisted of brain-related activity in which artifact components have been largely removed by filtering, ASR, ICA, and ICLabel. Each trial consisted of channel data from 4 to 28 s. In total there were 21 trials for each the Scale and Improv conditions. Leave-one-out cross-validation and Shuffle Split cross-validation were used to train and test the models. Therefore, different models are trained for each cross-validation step for each procedure. For shuffle split cross-validation, 100 iterations were used. The test size was 25% for each of the iterations (default parameters of the Python library scikit learn, open-source Python software). Separate machine learning models were trained for each participant. For leave-one-out cross-validation, statistical significance was determined by the Wilcoxon signed-rank test. The performance of the Shuffle Split cross-validation was used to demonstrate consistency with the classification performance of the leave-one-out cross-validation method.

The steps used for machine learning were as follows:

- For all machine-learning models, the channel level brain-related EEG was used as initial input for supervised training of CSP filters. Seven separate CSP filters were trained for the 7 frequency bands: theta (6.5–8 Hz), alpha1 (8.5–10 Hz), alpha2 (10.5–12 Hz), beta1 (12.5–18 Hz), beta2 (18.5–21 Hz), beta3 (21.5–30 Hz), and gamma (30.5–50 Hz). The input for training the leave-one-out cross-validation models consisted of 41 trials and their labels. The test set consisted of the one trial left out. In total 42 models were trained. One model for each trial left out for testing. The input for training the shuffle split cross-validation models consisted of a random selection of 75% of the whole dataset (33 trials) and their labels. The test set consisted of the remaining 25% of the dataset. In total there were 100 iterations of the shuffle split procedure. The CSP filters of the training set were applied to the respective test set.
- The CSP analysis generates the same number of filters as there are channels arranged in order from the maximal representation of condition 1 (improv) on one side to maximal representation of condition 2 (scale) on the other. We selected the two CSP filters maximally representing the two conditions. This serves to reduce the data from 64 channels to four CSP channels/components. This was done for all seven frequency bands.
- The PSD was calculated for each trial for training and test datasets within the corresponding frequency band of the CSP transformed data (four channels/components).
- The features used for training and testing the classification models consisted of the mean PSD value in each of the seven frequency bands for each of the four CSP filter channels/components (28 features in total; see Supplementary Figure S2 for the CSP weights for the frequency bands used for machine learning).
- Logistic regression models were used for the classification of improv and scale trials (Tomioka and Aihara, 2007; Tomioka et al., 2007; Blankertz et al., 2008).

## Results

### Source Localization of Brain Related Activity

**Figure 2:**
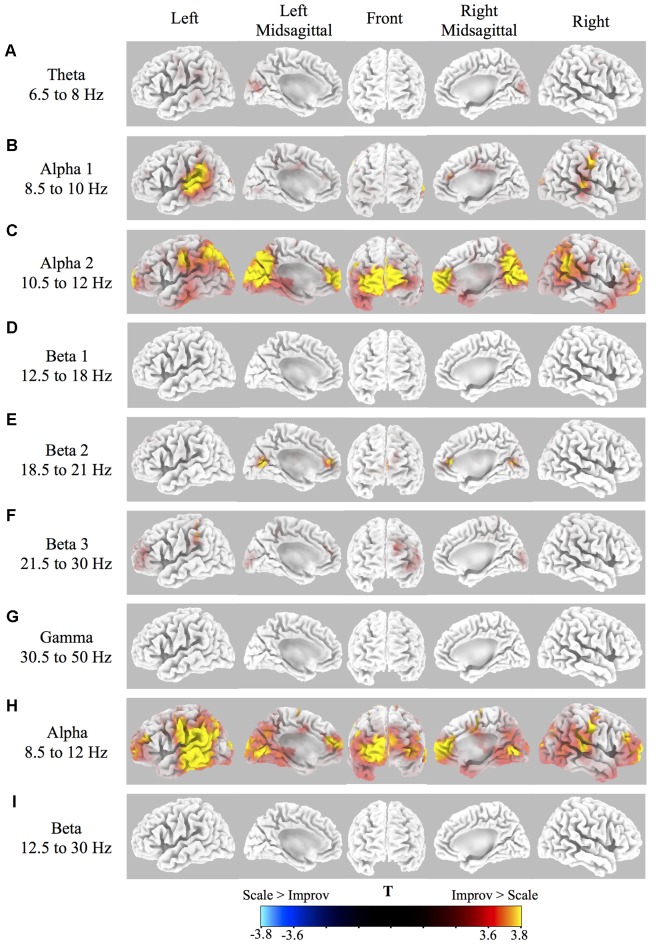
Cortical spectral power differences in various frequency bands for Improv > Scale. Source localization was conducted by sLORETA in the following frequency bands: **(A)** Theta 6.5–8 Hz; **(B)** Alpha1 8.5–10 Hz; **(C)** Alpha2 10.5–12 Hz; **(D)** Beta1 12.5–18 Hz; **(E)**. Beta2 18.5–21 Hz; **(F)** Beta3 21.5–30 Hz; **(G)** Gamma 30.5–50 Hz; **(H)** Full Alpha 8.5–12 Hz; **(I)** Full Beta 12.5–30 Hz. Statistically significantly differential power (Threshold for *p* < 0.05 correcting for multiple comparisons by SnPM = *T* > 3.467 two-tailed) for improv > scale is shown rendered on the surface of the cortex from yellow (high) to red (above threshold). No significant differential activity was present for the contrast of scale > improv.

Source localization of brain-related activity on the surface of the cortex (sLORETA) for the random effects group analysis of improv vs. scale is given for each frequency band (theta, alpha1, alpha2, beta1, beta2, beta3, gamma, full alpha, full beta) in Figure 2 and Table 1. Statistical non-parametric mapping analysis was used with 5,000 randomizations (LORETA Key software). Corrected critical thresholds and *p*-values for multiple comparisons were used in accordance with SnPM (Threshold for corrected *p* < 0.05 = *T* > 3.467 two-tailed; Nichols and Holmes, 2001). Statistically significant differences were only found for the improv > scale contrasts. There was no statistically significant differential activity for scale > improv in any of the frequency bands. Because sLORETA is a low-resolution technique the sources determined span large regions of the brain often spreading across several anatomical brain areas. This limitation should be kept in mind when making assertions about a specific discretely localized brain region.

**Table 1:** Peak coordinates of sLORETA source localization for various frequency bands.

Frequency band	Brain region	Peak MNI coordinates	*T*-value
Theta	Occipital Lobe, Cuneus BA17, 18	−15, −85, 15	4.05
Theta	Occipital Lobe, Cuneus BA17, 18	15, −85, 15	3.52
Alpha 1	MFC, ACC, BA 9, 32	5, 40, 25	3.92
Alpha 1	PreCG, PostCG, BA4, 3, 2	50, −15, 40	4.00
Alpha 1	STG, BA22, 41, 42	70, −25, 5	3.79
Alpha 1	STG, MTG, Insula BA22, 21, 41, 42, 39, 13	−45, −40, 20	4.03
Alpha 1	SMG, IPL, TPJ BA40	−55, −40, 30	3.96
Alpha 1	SPL, Precuneus, BA7	−25, −50, 50	3.75
Alpha 1	Superior Occipital Gyrus, BA17, 18	−20, −90, 20	3.76
Alpha 1	Middle Occipital Gyrus, BA18	30, −95, 15	3.76
Alpha 2	MFC, polar MPFC, SFG, ACC, BA10, 9, 32	10, 55, 15	4.44
Alpha 2	SMA, BA6	10, −10, 70	3.58
Alpha 2	PreCG, PostCG, PMC, BA4, 2, 3, 6	−55, −15, 35	3.94
Alpha 2	PreCG, PostCG, BA4, 2, 3	55, −15, 35	3.68
Alpha 2	ITG, MTG, STG, BA20, 21, 22	−65, −25, −20	3.69
Alpha 2	STG, BA22, 41, 42	60, −30, 15	3.72
Alpha 2	IPL, TPJ, BA40	−40, −35, 40	3.87
Alpha 2	IPL, TPJ, BA40	55, −50, 45	3.77
Alpha 2	Occipital Lobe, Cuneus, BA17, 18	−20, −90, 20	4.72
Alpha 2	Occipital Lobe, Cuneus, BA17, 18	5, −75, 10	4.33
Alpha 2	Precuneus, BA7	5, −65, 40	4.01
Beta 2	MFC, polar MPFC, ACC, BA10, 32	5, 40, 20	3.95
Beta 2	SPL, BA7	25, −70, 50	3.63
Beta 2	Precuneus, Cuneus, Post Cingulate, BA 31, 17, 18	0, −70, 15	4.03
Beta 3	SFG, MFC, BA10, 9	−15, 60, 25	3.63
Beta 3	DLPFC, MFG, BA10, 46, 45	−45, 45, 15	3.61
Beta 3	SMG, IPL, BA40	−55, −40, 40	3.75
Beta 3	Cuneus, BA17, 18	10, −85, 10	3.58
Alpha	MFC, ACC, SFG, BA9, 10, 32	5, 45, 20	4.16
Alpha	MFG, BA10	−35, 55, 10	3.77
Alpha	SFG, BA10	30, 60, 5	3.95
Alpha	PMC, BA6	−30, −10, 65	3.75
Alpha	Cingulate, BA24	5, −5, 40	3.71
Alpha	PreCG, PostCG BA4, 2, 3	−50, −15, 45	4.02
Alpha	PreCG, PostCG BA4, 2, 3	55, −15, 45	3.80
Alpha	STG, MTG BA22, 21, 41, 42	−65, −25, 0	3.97
Alpha	SMG, AG, IPL, SPL, BA40, 39, 7	−35, −65, 35	4.02
Alpha	STG, MTG, BA22, 21, 41, 42	60, −25, 10	3.81
Alpha	Precuneus, BA7	−25, −50, 50	4.07
Alpha	Occipital Lobe, Cuneus, BA17, 18	−20, −90, 20	4.58

*BA, Brodmann Area; MFC, Medial Frontal Cortex; ACC, Anterior Cingulate Cortex; PreCG, Precentral Gyrus; PostCG, Postcentral Gyrus; STG, Superior Temporal Gyrus; MTG, Middle Temporal Gyrus; SMG, Supramarginal Gyrus; TPJ, Temporal parietal junction; IPL, Inferior parietal lobule; SPL, Superior Parietal Lobule; MPFC, medial prefrontal cortex; SFG, Superior Frontal Gyrus; SMA, Supplementary motor area; ITG, Inferior Temporal Gyrus; MFG, Middle Frontal Gyrus; PMC, Premotor cortex; AG, Angular Gyrus. Negative × MNI coordinates are in left hemisphere and positive are in right hemisphere*.

The sLORETA results in the different frequency bands for improv vs. scale are as follows: the theta band frequency range showed significantly greater activity in the left and right cuneus in the occipital lobe (Figure 2A, Table 1). The alpha1 frequency range showed significant greater activity primarily in the left and right STG with greater activity on the left side also encompassing the Supramarginal Gyrus (SMG) and IPL with an additional cluster in the left Superior Parietal Lobule (SPL; Figure 2B, Table 1). Greater activity was also found in MFC (extending into anterior cingulate cortex, ACC), the right Precentral Gyrus (PreCG; and PostCG), as well as the left and right occipital gyrus (Figure 2B, Table 1). The alpha2 frequency range showed significantly greater activity in the MFC extending to the polar MPFC, Superior Frontal Gyrus (SFG), and ACC (Figure 2C, Table 1). Significantly increased activity in the alpha2 frequency range was also present in the supplementary motor area (SMA), left and right PreCG (and PostCG), the left and right STG, the left and right IPL, as well as the left and right cuneus (extending into precuneus; Figure 2C, Table 1). No significant differential activity correcting for multiple comparisons was found for the beta1 frequency range (Figure 2D). The beta2 frequency range showed significantly greater activity in the MFC extending to the polar MPFC, and ACC (Figure 2E, Table 1). Differential activity in the beta2 frequency range was also present in the SPL as well as the precuneus (extending into the cuneus and posterior cingulate (Figure 2E, Table 1). The beta3 frequency range showed significantly greater activity in the left DLPFC, the left SFG (extending into MFC), the left SMG, and the left and right cuneus (Figure 2F, Table 1). No significant difference in activity correcting for multiple comparisons was found for the gamma frequency range (Figure 2G). The full alpha range frequency band (8–12 Hz) had activity in the same regions as combining alpha1 and alpha2 bands (see above, as well as Figure 2H and Table 1). In contrast to the significant results for sub-bands, no significant differential activity correcting for multiple comparisons was found for full beta frequency range (12.5–30 Hz; Figure 2I).

### Machine Learning Analysis of Individual Participants

Machine learning employing logistic regression for the classifier, and CSP for the feature extraction over EEG recorded brain activity, was used to determine classification performance of whether the participant was playing scale or improv. Brain related features for each trial were extracted using CSP analysis separately in each of the seven frequency bands (theta, alpha1, alpha2, beta1, beta2, beta3, gamma). Four CSP filters were trained to separate scale from improv trials (for details, see “Materials and Methods” section). The classification performance was determined using a leave-one-out cross-validation procedure. Therefore, different models are trained for each cross-validation step. In total there were 42 cross-validation steps, one for each of the 21 scale and 21 improv trials. Separate analyses were conducted for each participant. The results of the machine learning performance (percent correct classification, hit rate, false alarm rate, d-prime) for classifying scale vs. improv are given in Table 2 for each participant. The Wilcoxon signed-rank test revealed that 11 of the 14 participants had classification performance that was significantly better than chance level (Table 2). The mean classification performance of all participants was 75.75% (SE = 3.49), which was significantly greater than the chance level (Wilcoxon signed-rank *p* < 0.05). A shuffle split cross-validation procedure (100 iterations) was also employed to verify the robustness of the classification performance. The overall mean classification performance for the shuffle split cross-validation procedure (74.76%; SE = 3.10; Wilcoxon signed-rank *p* < 0.05) was in close agreement to that of the leave one out cross-validation procedure (75.75%).

**Table 2:** Machine learning results: logistic regression (Leave-One-Out Cross Validation).

Participant	Percent correct	Hit rate	False alarm rate	d-prime
01	81.95*	0.810	0.190	1.752
02	76.19*	0.810	0.286	1.442
03	76.19*	0.750	0.227	1.422
04	83.33*	0.800	0.136	1.938
05	61.91	0.619	0.381	0.606
06	76.19*	0.810	0.286	1.442
07	80.95*	0.857	0.238	1.780
08	61.91	0.667	0.429	0.611
09	64.29*	0.667	0.381	0.734
10	90.48*	0.905	0.095	2.618
11	83.33*	0.850	0.182	1.945
12	92.86*	0.952	0.095	2.978
13	47.62	0.429	0.476	−0.120
14	83.33*	0.810	0.143	1.944
Mean	75.75*	0.767	0.253	1.507
SE	3.49	0.037	0.035	0.230

*Classification performance percent correct statistically greater than chance (50%) *p* < 0.05 using Wilcoxon signed-rank test.

The mean weights across the 42 trained models within each participant were evaluated to determine the regularities and individual differences across participants with regards to the relative contribution of different frequency bands to classification accuracy. The mean CSP filters (two for scale and two for improv) for each frequency band (28 total CSPs) for each participant are given in Supplementary Figure S1. There were considerable individual differences in the CSPs across all frequency ranges and their relative weighting by the logistic regression model across participants (Supplementary Figure S2). The six top weights of the within-participant mean machine learning classification model are surrounded by a black square (Supplementary Figure S2). Because the polarity of the model weight is in relation to the CSP, which can be arbitrarily flipped, the absolute magnitude of the weight from zero was used to determine its contribution. For each frequency band (consisting of four CSP filters) the percentage of participants that had at least one of the top six weights in that band is as follows (sorted in descending order of prevalence): Theta = 79%; Gamma = 79%; Beta2 = 71%; Alpha1 = 64%; Alpha2 = 64%; Beta3 = 64%; Beta1 = 36%;. To further understand the importance of different frequency bands in predicting improvisation, the contribution of the largest absolute model weight within each frequency band across participants was evaluated. The mean maximum absolute weight in each frequency band across participants was as follows: Theta = 0.67 (SE = 0.07); Alpha1 = 0.47 (SE = 0.05); Alpha2 = 0.56 (SE = 0.06); Beta1 = 0.34 (SE = 0.03); Beta2 = 0.50 (SE = 0.05); Beta3 = 0.52 (SE = 0.06); Gamma = 0.70 (SE = 0.09). The Theta band was significantly greater than the Alpha1, Beta1, and Beta2 bands; The Alpha1 band was significantly greater than the Beta1 band; The Alpha2 band was significantly greater than the Beta1 band; The Beta1 band was not significantly higher than any bands; The Beta2 band was significantly higher than the Beta1 band; The Beta3 band was significantly higher than the Beta1 band; The gamma band was significantly higher than the Alpha1 and Beta1 bands (Wilcoxon signed-rank *p* < 0.05).

## Discussion

In this study, we investigated brain-related EEG activity in professional and amateur guitar players who are skilled at improvisation. The experimental contrast of improv vs. scale was used to probe brain regions differentially involved with guitar improvisation. The two tasks, improvisation, and scale, differed considerably with respect to their creative demands. In the improvisation task, participants were instructed to perform in a manner that was as original and creative as possible. In contrast, in the scale task, participants were asked to play the major scale (a common standard scale that is a sequence of completely predictable notes). The scale condition consisted of a non-creative task that controlled for general aspects of motor control and sensory aspects of guitar playing.

Our research helps to better validate EEG findings into the investigation of brain activity underlying improvisation by utilizing advanced techniques (ASR, ICA and ICLabel) to clean and exclude artifacts from the data. Although body movement is an inherent part of playing a musical instrument, previous EEG research investigating improvisation has not used techniques such as ICA to separate brain activity from physiological and movement-based artifacts (Dolan et al., 2013, 2018; Müller et al., 2013; Wan et al., 2014; Dikaya and Skirtach, 2015; Adhikari et al., 2016; Sanyal et al., 2016; Lopata et al., 2017).

### Brain Related Activity Differentiating Improv and Scale

Consistent with our predictions based on previous EEG/MEG and fMRI research significant differential power for improv > scale was found in brain regions involved with internally mediated cognitive processing (MFC, polar MPFC), externally directed cognitive control (DLPFC, IFG), and perceptual-motor planning/control (STG, IPL, SPL, TPJ) in the alpha (Figures 2B,C,H, Table 1) and beta (Figures 2E,F, Table 1) frequency ranges.

It is thought that increased activity in the MFC may denote internally directed attention involved with creativity tasks as well as coordination of complex action underlying sequential planning that is an integral part of improvisation of music (Dolan et al., 2013; Beaty, 2015; Dikaya and Skirtach, 2015; Landau and Limb, 2017; Stevens and Zabelina, 2019). Our finding of greater power for improv over scale in the frontal area comprising regions of the MFC, ACC, and SMA (Figure 2, Table 1) are consistent with many fMRI studies investigating improvisation (Bengtsson et al., 2007; de Manzano and Ullén, 2012a, b; Donnay et al., 2014; Pinho et al., 2014; McPherson et al., 2016; Landau and Limb, 2017; Lu et al., 2017; Dhakal et al., 2019). The low alpha range has been implicated with general attentional demands and the upper alpha band to task-specific demands often related to creativity (Fink and Benedek, 2014). The beta frequency range has also been divided into subbands based on different responses to varying tasks (Kropotov, 2016). Differences within this various alpha and beta subbands in our study suggest that they may represent distinct underlying processes.

It should be pointed out that alpha frequency band power is often thought to reflect cortical inhibition (cortical idling) in various brain regions (e.g., DLPFC, occipital regions; Fink and Benedek, 2014; Dikaya and Skirtach, 2015; Stevens and Zabelina, 2019). This, however, is not always the case. The alpha frequency range has been reported to be involved with cortical excitation in the MFC in tasks involving creative processing and improvisation consistent with the conclusions of our study (Fink and Neubauer, 2008; Jauk et al., 2012; Benedek et al., 2014; Schwab et al., 2014; Lopata et al., 2017; Camarda et al., 2018; Lopata et al., 2017; Stevens and Zabelina, 2019). Furthermore, studies using fMRI and EEG have shown increased alpha synchronization in frontal and parietotemporal brain regions associated with brain activation during creative processing (Fink and Neubauer, 2008).

Also of particular interest is the finding of significantly greater power for improv over scale in the polar MPFC in the alpha2 and beta2 frequency range (Figures 2C,E, Table 1). The polar MPFC is thought to be involved with the production of spontaneous internally motivated cognition (self-expression) and has been found to be active during music improvisation (Limb and Braun, 2008; Liu et al., 2012; Landau and Limb, 2017). Because the MPFC is very close to the eyes it is possible that this activity may reflect differences in eye movement and blink activity between the improv and scale conditions. However, we do not believe this is the case in our experiment because ICA was able to extract eye related artifacts for all participants in this study.

Activations in the polar MPFC together with deactivation in DLPFC (involved with executive control mediated by conscious self-monitoring with directed attention) are thought to reflect neural correlates of “flow” (Limb and Braun, 2008; Landau and Limb, 2017). Flow is thought to be an effortless conscious state characterized by internally motivated actions with minimal top-down control (Limb and Braun, 2008; Landau and Limb, 2017).

While we did find a significantly greater power in polar MPFC we did not find a deactivation in DLPFC during improvisation. Rather, in our study we found the opposite pattern of activity. There was significantly greater power in the left DLPFC in the Beta3 frequency range (Figure 2F, Table 1). Our finding of greater power in the beta band for improv is not consistent with the results of Boasen et al. (2018), which showed a decrease in the beta band power in the rostral MFC including the DLPFC for improvisation relative to a control condition in a MEG experiment. Additionally, many fMRI studies also revealed that improvisation tasks compared to respective control conditions showed significant deactivations in DLPFC (Limb and Braun, 2008; Liu et al., 2012; Landau and Limb, 2017; Boasen et al., 2018). Despite this, many other studies have also found increases in the DLPFC region consistent with our finding of increased beta power in this region (Figure 2F, Table 1; Bengtsson et al., 2007; de Manzano and Ullén, 2012a, b; Beaty, 2015). One potential reason for the discrepancy in results between studies may be related to the skill level of the participants. It has been put forward that a decrease in activity in this region is associated with less executive control and top-down processing related to the concept of “flow” for advanced improvisation musicians (Limb and Braun, 2008; Landau and Limb, 2017). The differences in DLPFC activity across studies may be explained by the degree of automatic vs. controlled processes (Beaty, 2015; Sowden et al., 2015; Rosen et al., 2016) involved that are dependent on expertise. Musicians that have not reached a high skill level may require more directed coordination of motor activity that is mediated by executive control. Therefore, while highly skilled improvisation musicians would be expected, based on past results, to show a decrease in activity in executive control areas such as the DLPFC, more novice improvisation musicians, such as those in this study, would be predicted to have greater activity in DLPFC associated with explicit top-down control. Support of this distinction comes from a study in which anodal transcranial direct current stimulation tDCS to the right DLPFC enhanced improvisation performance in novice musicians but degraded improvisation performance in expert musicians (Rosen et al., 2016).

It has further been suggested the improvisation, as well as other creative processes, involves interplay between brain networks involved in externally directed attention (such as the DLPFC) and those involving internally directed attention (polar MPFC, MFC; Beaty, 2015). Many of the differences in activation of brain regions involved with externally directed attention (e.g., DLPFC) across studies investigating improvisation have been attributed to differences in the degree of constraints for the experimental task (Berkowitz and Ansari, 2010; de Manzano and Ullén, 2012a). Studies (e.g., Limb and Braun, 2008) that were relatively unconstrained showed deactivation of brain regions involved with externally directed attention (e.g., DLPFC), whereas studies with greater external constraints on improvisation, including playing to a metronome and playing in collaboration with a second performer, showed greater activity in brain regions involved with externally directed attention (Bengtsson et al., 2007; Berkowitz and Ansari, 2008; de Manzano and Ullén, 2012a; Donnay et al., 2014). Our study shows activity in brain regions involved with internally directed attention (polar MPFC, MFC) as well as those involved with externally directed attention (DLPFC).

There was considerably greater power for improv over scale in visual areas across many of the frequency bands investigated in this study (theta, alpha1, alpha2, beta2, beta3, and alpha; Figures 2A–C,E,F,H; Table 1). This activity was located in the cuneus and precuneus regions as well as other visual processing regions in the occipital gyrus. One possible explanation for greater activity in these regions is the greater need for visual observation of the fingers and hands in relation to the guitar for improvisation overplaying scales. This is consistent with demands for externally directed attention.

Another interesting finding in our study is the presence of significantly greater power for improv over scale in the alpha frequency range in brain regions including the PMC, STG, IPL, and the TPJ (Figures 2B,C,H, Table 1) involved with perceptual-motor planning and control as well as feedback regulation based on external and internal states and goals. In music processing, these regions have been found to be involved in manipulations of musical structures (Zatorre et al., 2010), and motor-auditory interactions mediated through the parietal cortex has been suggested to be required for musical rhythm perception and production (Iversen and Balasubramaniam, 2016). These functions are thought to be indicative of processes involved with music improvisation. Activation of the PMC is consistent with the study of Berkowitz and Ansari (2008) suggesting the role of this area in movement coordination and sequence generation in relation to music improvisation. The STG/sulcus STG/S is an auditory processing region that is likely differentially engaged during improvisation to monitor the ongoing acoustic environment. The IPL is involved with sensory predictive feedback (Pecenka et al., 2013). The TPJ is thought to mediate transformation of auditory signals into a form that constrains motor processing (Griffiths and Warren, 2002; Warren et al., 2005; Callan et al., 2006) and is known to be involved with reward-based modulation by music (Li et al., 2015). It should be noted that right TPJ was found to show decreased activity in expert musicians during improvisation in the study by Berkowitz and Ansari (2010), contrary to the left TPJ increase found in our study. It is hypothesized by Berkowitz and Ansari (2010) that a decrease in activity in TPJ for expert musicians results from inhibition of stimulus-driven attention. In the non-elite musicians used in our experiment the results suggest that improvisation utilizes a network of brain regions involved with coordinating planned sequences of movement that are modulated in response to both ongoing environmental context through monitoring and feedback of sensory states in relation to internal plans and goals. It is interesting to point out that a network between brain regions involved with cognitive control (IFG) and those with low-level imaginative processes (default mode network; which includes the MFC and IPL regions found to be active in our study) is implicated in the generation of creativity (Beaty et al., 2014). However, this form of creativity may be different from that used by expert improvisers that experience a state of flow that is characterized by less cognitive control and self-monitoring (Landau and Limb, 2017).

We believe it is unlikely that our results are merely due to basic aspects of acoustics that may differ between improv and scale. With respect to the acoustics, no amplifier was used, the volume of the notes played on the guitar, although audible, were considerably less than the sound from the metronome and the context chord sound presented. Therefore it is unlikely that mere overall acoustic intensity is responsible for activity in auditory regions found in our study. Rather, our results of activity in the left STG are consistent with attentional modulation to focus on music generated as well as background context in the service of producing future notes in accordance with improvisation.

It is also unlikely that our results are merely due to basic aspects of motor activity, such as performance rate, that may differ between improv and scale. Several studies have reported brain activity in primary sensorimotor cortices (PreCG and PostCG), SMA, PMC, IPL, basal ganglia, and anterior cerebellum as a result of the rate of finger tapping movement (Witt et al., 2008). While many of these regions (Figure 2, Table 1) were found to show greater power in improv in our study we suggest that this is not a result of guitar playing rate differences between improv and scale conditions. This is because although variable pace was an option in the improv condition, all participants but two, played at the same eighth-note rate as was required for the scale condition. The two subjects that played improv in some trials with a different beat performed at a lower rate (quarter note). Therefore it is unlikely that the differences we found in these brain regions were merely a result of different rates or different rhythms in the two conditions but are more likely related to complex coordinated perceptual-motor control in relation to internal goals involved with improvisation.

Future research will include a precise measure of the acoustics and finger movements synchronized to the recording of brain activity. In this way, we can conduct time-frequency analyses over the source localized brain activity and use these variables to address potential confounds such as finger movement and acoustic variables while at the same time trying to determine temporal and spectral correlates of neural processes underlying improvisation.

### Machine Learning

An additional goal of our research was to determine if individual-specific brain-related activity could be used to classify whether the musician is playing improv or scale using machine-learning techniques. We used bandpass filtering and CSP to extract features and a conventional machine learning method (logistic regression) to confirm that we can classify these two different tasks from brain-related activity. It is important to note that this data consists of brain-related activity in which artifact components have been largely removed by ASR, ICA, and ICLabel. The use of machine learning to classify improv vs. scale is an important step towards developing a brain-computer interface that can possibly be used for neurofeedback training.

Using a leave-one-out cross-validation testing procedure the performance could be evaluated using a separate model for each trial. The mean classification performance across participants was 75.75% and was significantly greater than chance (Table 2). The robustness of these results was evaluated using a shuffle split cross-validation procedure which achieved a similar mean classification performance of 74.76%. Individual participant classification performance ranged from 47.62% to 92.86% (Table 2). Better than chance classification performance was present for 11 of the 14 participants (Table 2). These results show that for at least for some participants very good models can be trained to classify improv from scale trials using seven frequency bands and four CSP spatial filters for a total of 28 features.

The relationship between the weighting of the features by the trained logistic regression models was explored. One advantage of using logistic regression over other machine learning classification methods is that the weights of the various features used for classification can be assessed. An attempt was made to determine if there were any cross-individual regularities in the weighting of certain frequency bands (out of the seven used: Theta, Alpha1, Alpha2, Beta1, Beta2, Beta3, Gamma). The weights with the top six absolute values of the features were determined (see squares around CSP filters in Supplementary Figure S2). The frequency bands that had the largest number of participants with high weights were theta and gamma with 11, followed by Beta2 with 10, then Alpha1, Alpha2, and Beta3 with nine and the lowest were Beta1 with five. To better evaluate these regularities in frequency band weights across participants, statistics were carried out across the maximum absolute weight within each frequency band. The results confirmed that Theta and Gamma frequency bands were in general weighted more highly than many of the other frequency bands (see “Results” section). Gamma band activity is thought in part to be involved with bottom-up and top-down information matching, which may be important for improvisation (Stevens and Zabelina, 2019). These results are interesting in that they are different than what one would expect given the results of the sLORETA analysis (Figure 1, Table 2), which showed no significant differential activity in the Gamma range (However a significant differential activity was shown in the theta range). One likely explanation for this discrepancy is the degree of individual differences in the location of gamma activity in the brain across participants (Supplementary Figure S2 shows considerable differences in the CSP filters for the participants in all frequency ranges).

### Limitations

While our study did control for general aspects of finger and hand movement as well as general acoustic variables it did not control for the musical complexity of the improvisation. It is possible that brain differences between the improv and scale condition are a result of perceptual processing related to the complexity of the music rather than to processes solely related to the creative processes underlying improvisation. Previous studies have shown that creativity including improvisation involves many brain networks encompassing frontal, parietal, and limbic brain regions known to be involved with multiple functions such as executive processing, attention, memory, and emotion (Fink et al., 2009a; Beaty, 2015; Landau and Limb, 2017; Beaty et al., 2018). It is unlikely that there is a specialized area of the brain that is solely dedicated to improvisation and creative processing alone. While our study can determine whether there is significant differential activity in various brain regions between improvisation and the control task of playing scales it cannot discern whether these localized brain processes are specific to improvisation. This is partly because of the necessity to exclude the rest condition from the analysis because of excessive artifacts. The rest condition could have been used as a baseline condition for comparison with the experimental conditions so that one could tell the degree of activity for each condition separately.

It should be pointed out that common to many creative skills there is natural variation in ability across individuals that is often difficult to quantify. Because of this, the performance level across participants is not constant. This is a common limitation to group analysis that is present in all performance-related research. This is true especially in the case in which performance quality is subjective. In the future we intend to extend this research to investigate differences in neural processes between skilled and novice improvisation as a way to study skill learning and resolve some of the discrepancies in the literature, which we speculated above may be due to skill level.

Another limitation of our study was the need to remove many bad channels and then interpolate for some of the participants (good and bad electrodes are shown in Supplementary Figure S1). Because this study was a within-subjects design it is unlikely that the differences found between the improv and scale conditions are a result of bad channels and interpolation as they were identical for each condition. There is, however, a likely bias in the identified sources based on the electrode density with respect to interpolation. It is known that shallow sources are more prone to interpolation errors than deep sources (Fletcher et al., 1996). With respect to topographic maps (as used by ICLabel for identifying brain-related components), it is known that interpolation error will occur when there is a high potential gradient far from any electrode (Fletcher et al., 1996). Inaccuracies in the topo maps may limit the accuracy upon which ICLabel selects brain and artifact related ICs. Based on an analysis of the location of bad channels showing they are mostly in posterior regions (central parietal and parietal occipital channels; see “Materials and Methods” section and Supplementary Figure S1) it is likely that interpolation and source localization error is greater in these regions for our study. Whereas anterior and frontal central regions are likely to have lower interpolation and source localization error due to the presence of fewer bad channels in our study.

For three of the participants, there were a considerable number of bad channels (see Supplementary Figure S1; Participants P02, P03, and P07). To help ensure that the results of the sLORETA analysis are not biased by potential interpolation error these three participants were removed from the random effects analysis of improv relative to the scale. The result of this analysis is given in Supplementary Figure S3. Although the T threshold is lower in the analysis with the three participants excluded it does show good consistency with the original results shown in Figure 2 using all 14 participants. Correction for multiple comparisons separately in alpha (threshold *p* < 0.05 = *T* > 3.37) and beta (threshold *p* < 0.05 = *T* > 3.23) frequency bands for the 11 participant analysis does reveal considerable significant activity that is also present for the full 14 participant analysis (compare Supplementary Figure S3 and Figure 2). The consistency of these results and the similarity of the location of brain activity with several fMRI studies using a similar experimental paradigm lend support to the validity of our findings and suggest that they are not likely entirely due to interpolation error.

We relied on ICLabel to identify brain ICs, rather than our subjective interpretation of the data component topographies, leading to the inclusion of some components with the appearance of single-channel topographies. ICLabel is a tool trained to identify component type based on spectrotemporal features of thousands of example components scored by expert scientists and has been demonstrated to have expert-level performance on test data (Pion-Tonachini et al., 2019). As a check on the ICLabel results, we conducted a dipole analysis using DIPFIT (EEGLAB). Dipole analysis localized nearly all of the brain-related components identified by ICLabel within the volume of the brain, thus corroborating to a large degree the ICLabel results. Exceptions were five components (out of a total of 67 across all 14 participants) for three participants that were located on the skull instead of within the volume of the brain (P01 component 1; P04 components 2, 4, 6; P09 component 1). The reason we chose the more holistic ICLabel approach is that single equivalent dipole analysis may be limited when dealing with multiple and/or distributed sources. This is one of the reasons for using sLORETA analysis in order to describe distributed source localization.

There are several studies that have demonstrated that a few and even a single IC can be used for sLORETA analysis (Grau et al., 2007; Slobounov et al., 2009; Ventouras et al., 2010). In this respect the low number of brain-related ICs retained in a few of the participants is not problematic. It is possibly biased by the conservative criteria we set for ICLabel but this is a tradeoff with corruption of the data by potential artifacts. Furthermore, the comparison of improv vs. scale is made independently for each participant based on using the same number of components for the sLORETA analysis. One potential bias that may arise from a low number of ICs being used for some participants and not others is that they might be missing activity in various brain regions that could be task-related. Critically, this bias is conservative, working against our hypothesis and is likely to result in the absence of finding a significant difference between conditions (false-negative, Type II error), rather than a false positive, Type I error. Therefore, the results found to be significant in our study are not likely biased by the low number of brain ICs found for some participants. Note, we do not claim that our ICs represent all of the brain activity of a participant, but only that which can confidently be attributed to neural, and not artifactual, processes.

It should be recognized that ICLabel along with the other automated preprocessing steps utilized to minimize the degree of artifacts in the data are open to improvement but have the advantage of total replicability due to the removal of subjective choices in the processing stream.

## Conclusion

This study investigated neural processes related to playing improvisation relative to scale music. It was our goal to extend EEG research related to brain processes underlying music improvisation by using high-density EEG recording (64 channel), cortical source localization, advanced artifact extraction, and machine learning techniques. The results of this research revealed that improvisation over scale is characterized by increased power in theta, alpha, and beta frequency range in brain regions involved with internally directed attention and control (MFC, polar MPFC), those involved with externally directed attention (DLPFC), as well as those involved with feedback regulation of perceptual-motor planning and control based on external and internal states and goals (PMC, STG, IPL, TPG, PreCG, PostCG), thought to be important for execution of improvisation. Converging findings from both fMRI and EEG studies using various musical instruments and tasks suggest that these results are generalizable to different modalities and give support for the conclusion that they underlie creative processes used for improvisation. A long-term goal is to be able to use neurofeedback and neuromodulation methods to develop neuroergonomic based technology to improve improvisation training and performance. One could envision a brain-computer-interface that could give rewards as feedback when certain optimal improvisation-based brain states are present. Alternatively, a neuroadaptive interface could be utilized to signal the performer when to start improvisation based on ongoing brain activity.

## Data Availability Statement

The raw data supporting the conclusions of this article will be made available by the authors, without undue reservation, to any qualified researcher.

## Ethics Statement

This study was carried out in accordance with the recommendations of National Institute of Information and Communications Technology (NICT) Human Subject Review Committee with written informed consent from all subjects. All subjects gave written informed consent in accordance with the Declaration of Helsinki. The protocol was approved by the National Institute of Information and Communications Technology (NICT) Human Subject Review Committee.

## Author Contributions

MS and DC designed and conducted the experiment and analyzed the data. MS, JI and DC wrote the manuscript.

## Conflict of Interest

The authors declare that the research was conducted in the absence of any commercial or financial relationships that could be construed as a potential conflict of interest.

## Footnotes

^1^https://sccn.ucsd.edu/wiki/Makoto’s_preprocessing_pipeline

^2^https://sccn.ucsd.edu/wiki/ICLabel

## Supplementary Material

FIGURE S1 Channel and IC information for each participant. The good channels, Black (included in the analysis), and bad channels, Red (removed from analysis), are shown in a topographic plot. The PCA reduction is the number of ICs to retain for ICA. The number is one less than the number of good channels to account for the loss of rank from the average referencing procedure. Topo maps of brain-related independent components ICs. The topographic mapping (topo map) of the scalp data field in a 2-D circular view (nose is at the top) for the brain related ICs determined by ICLabel are given for each of the 14 participants P01 to P14.

FIGURE S2 Topo maps of the weights of the common spatial pattern (CSP) filters used as features for machine learning. The features for each of the 14 participants are shown consisting of four CSP filters (two for Scale and two for Improv) for each frequency bands (Theta 6.5–8 Hz; Alpha1 8.5–10 Hz; Alpha2 10.5–12 Hz; Beta1 12.5–18 Hz; Beta2 18.5–21 Hz; Beta3 21.5–30 Hz; Gamma 30.5–50 Hz). The weights of the logistic regression machine learning classifier with the top six absolute values of the features are depicted by a black square around the topo map of the CSP filter.

FIGURE S3 Cortical spectral power differences in alpha and beta frequency bands for Improv > Scale with three participants with many interpolated channels removed. Source localization was conducted by sLORETA in the following frequency bands: **(A)** Alpha1 8.5–10 Hz; **(B)** Alpha2 10.5–12 Hz; **(C)** Beta2 18.5–21 Hz; **(D)** Beta3 21.5–30 Hz. Statistically significantly differential power (Threshold for *p* < 0.05 correcting for multiple comparisons separately in alpha (SnPM = *T* > 3.372 two-tailed) and beta (SnPM = *T* > 3.229 two-tailed) for improv > scale is shown rendered on the surface of the cortex from yellow (high) to red (above threshold). The results are similar to those reported in Figures 2B,C,E,F suggesting to some degree that the sLORETA results are not due to interpolation error.

## References

[B1] Adhikari B. M. Norgaard M. Quinn K. M. Ampudia J. Squirek J. Dhamala M. (2016). The brain network underpinning novel melody creation. Brain Connect. 6, 772–785. 10.1089/brain.2016.0453 27750434

[B2] Artoni F. Fanciullacci C. Bertolucci F. Panarese A. Makeig S. Micera S.. (2017). Unidirectional brain to muscle connectivity reveals motor cortex control of leg muscles during sterotyped walking. Neuroimage 159, 403–416. 10.1016/j.neuroimage.2017.07.013 28782683 PMC6698582

[B3] Beaty R. E. (2015). The neuroscience of musical improvisation. Neurosci. Biobehav. Rev. 51, 108–117. 10.1016/j.neubiorev.2015.01.004 25601088

[B4] Beaty R. E. Benedek M. Wilkins R. W. Jauk E. Fink A. Silvia P. J.. (2014). Creativity and the default network: a functional connectivity analysis of the creative brain at rest. Neuropsychologia 64, 92–98. 10.1016/j.neuropsychologia.2014.09.019 25245940 PMC4410786

[B5] Beaty R. Kenett Y. Christensen A. Rosenberb M. Benedek M. Chen Q.. (2018). Robust prediction of individual creative ability from brain functional connectivity. Proc. Natl. Acad. Sci. U S A 115, 1087–1092. 10.1073/pnas.1713532115 29339474 PMC5798342

[B6] Bell A. J. Sejnowski T. J. (1995). An information maximisation approach to blind separation and blind deconvolution. Neural Comput. 6, 1129–1159. 10.1162/neco.1995.7.6.1129 7584893

[B7] Benedek M. Borovnjak B. Neubauer A. C. Kruse-Weber S. (2014). Creativity and personality in classical, jazz and folk musicians. Pers. Individ. Dif. 63, 117–121. 10.1016/j.paid.2014.01.064 24895472 PMC3989052

[B8] Bengtsson S. Csíkszentmihályi M. Ullén F. (2007). Cortical regions involved in the generation of musical structures during improvisation in pianists. J. Cogn. Neurosci. 19, 830–842. 10.1162/jocn.2007.19.5.830 17488207

[B9] Berkowitz A. L. Ansari D. (2008). Generation of novel motor sequences: the neural correlates of musical improvisation. Neuroimage 41, 535–543. 10.1016/j.neuroimage.2008.02.028 18420426

[B10] Berkowitz A. L. Ansari D. (2010). Expertise-related deactivation of the right temporoparietal junction during musical improvisation. Neuroimage 49, 712–719. 10.1016/j.neuroimage.2009.08.042 19715764

[B11] Bigdely-Shamlo N. Mullen T. Kothe C. Su K. Robbins K. (2015). The PREP pipeline: standardized preprocessing for large-scale EEG analysis. Front. Neuroinform. 9:16. 10.3389/fninf.2015.00016 26150785 PMC4471356

[B12] Blankertz B. Tomioka R. Lemm S. Kawanabe M. Müller K. (2008). Optimizing spatial filters for robust EEG single-trial analysis. IEEE Signal Process. Mag. 25, 41–56. 10.1109/msp.2008.4408441

[B13] Boasen J. Takeshita Y. Kuriki S. Yokosawa K. (2018). Spectral-spatial differentiation of brain activity during mental imagery of improvisational music performance using MEG. Front. Hum. Neurosci. 12:156. 10.3389/fnhum.2018.00156 29740300 PMC5928205

[B14] Callan D. E. Durantin G. Terzibas C. (2015). Classification of single-trial auditory events using dry-wireless EEG during real and motion simulated flight. Front. Syst. Neurosci. 9:11. 10.3389/fnsys.2015.00011 25741249 PMC4330719

[B15] Callan D. E. Gateau T. Durantin G. Gonthier N. Dehais F. (2018). Disruption in neural phase synchrony is related to identification of inattentional deafness in real-world setting. Hum. Brain Mapp. 39, 2596–2608. 10.1002/hbm.24026 29484760 PMC6866488

[B16] Callan D. Tsytsarev V. Hanakawa T. Callan A. Katsuhara M. Fukuyama H.. (2006). Song and speech: brain regions involved with perception and covert production. Neuroimage 31, 1327–1342. 10.1016/j.neuroimage.2006.01.036 16546406

[B17] Camarda A. Salvia É. Vidal J. Weil B. Poirel N. Houdé O.. (2018). Neural basis of functional fixedness during creative idea generation: an EEG study. Neuropsychologia 18, 4–12. 10.1016/j.neuropsychologia.2018.03.009 29530800

[B18] Chang C.-Y. Hsu S.-H. Pion-Tonachini L. Jung T.-P. (2018). “Evaluation of Artifact subspace reconstruction for automatic EEG artifact removal,” in Conference Proceedings: Annual International Conference of the IEEE Engineering in Medicine and Biology Society (Honolulu, HI, USA: IEEE), 1242–1245. 10.1109/EMBC.2018.8512547 30440615

[B21] Delorme A. Makeig S. (2004). : EEGLAB: an open source toolbox for analysis of single-trial EEG dynamics including independent component analysis. J. Neurosci. Methods 134, 9–21. 10.1016/j.jneumeth.2003.10.009 15102499

[B19] de Manzano Ö. Ullén F. (2012a). Goal-independent mechanisms for free response generation: creative and pseudo-random performance share neural substrates. Neuroimage 59, 772–780. 10.1016/j.neuroimage.2011.07.016 21782960

[B20] de Manzano Ö. Ullén F. (2012b). Activation and connectivity patterns of the presupplementary and dorsal premotor areas during free improvisation of melodies and rhythms. Neuroimage 63, 272–280. 10.1016/j.neuroimage.2012.06.024 22732560

[B23] Dhakal K. Norgaard M. Adhikari B. M. Yun K. S. Dhamala M. (2019). Higher node activity with less functional connectivity during musical improvisation. Brain Connect. 9, 296–309. 10.1089/brain.2017.0566 30618291

[B24] Dikaya A. L. Skirtach I. (2015). Neurophysiological correlates of musical creativity: the example of improvisation. Psychol. Russ. State Art 8, 84–97. 10.11621/pir.2015.0307

[B25] Dolan D. Jensen H. J. Mediano P. A. M. Molina-Solana M. Rajpal H. Rosas F.. (2018). The improvisational state of mind: a multidisciplinary study of an improvisatory approach to classical music repertoire performance. Front. Psychol. 9:1341. 10.3389/fpsyg.2018.01341 30319469 PMC6167963

[B26] Dolan D. Sloboda J. Jensen H. Cruts B. Feygelson E. (2013). The improvisatory approach to classical music performance: an empirical investigation into its characteristics and impact. Music Perform. Res. 6, 1–38.

[B27] Donnay G. F. Rankin S. K. Lopez-Gonzalez M. Jiradejvong P. Limb C. J. (2014). Neural substrates of interactive musical improvisation: an fMRI study of ‘trading fours’ in jazz. PLoS One 9:e88665. 10.1371/journal.pone.0088665 24586366 PMC3929604

[B3011] Fink A. Benedek M. (2014). EEG alpha power and creative ideation. Neurosci. Biobehav. Rev. 44, 111–123. 10.1016/j.neubiorev.2012.12.002 23246442 PMC4020761

[B31] Fink A. Grabner R. H. Benedek M. Reishofer G. Hauswirth V. Fally M.. (2009a). The creative brain: investigation of brain activity during creative problem solving by means of EEG and fMRI. Hum. Brain Mapp. 30, 734–748. 10.1002/hbm.20538 18266217 PMC6871103

[B29] Fink A. Neubauer A. C. (2006). EEG α oscillations during the performance of verbal creativity tasks: differential effects of sex and verbal intelligence. Int. J. Psychophysiol. 62, 46–53. 10.1016/j.ijpsycho.2006.01.001 16503062

[B30] Fink A. Neubauer A. C. (2008). Eysenck meets Martindale: the relationship between extraversion and originality from the neuroscientific perspective. Pers. Individ. Dif. 44, 299–310. 10.1016/j.paid.2007.08.010

[B33] Fletcher E. M. Kussmaul C. L. Mangun G. R. (1996). Estimation of interpolation errors in scalp topographic mapping. Electroencephalogr. Clin. Neurophysiol. 98, 422–434. 10.1016/0013-4694(96)95135-4 8647046

[B34] Fuchs M. Kastner J. Wagner M. Hawes S. Ebersole J. S. (2002). A standardized boundary element method volume conductor model. Clin. Neurophysiol. 113, 702–712. 10.1016/s1388-2457(02)00030-5 11976050

[B35] Grau C. Fuentemilla L. Marco-Pallarés J. (2007). Functional neural dynamics underlying auditory event-related N1 and N1 suppression response. Neuroimage 36, 522–531. 10.1016/j.neuroimage.2007.03.027 17499521

[B36] Griffiths T. D. Warren J. D. (2002). The planum temporale as a computational hub. Trends Neurosci. 25, 348–353. 10.1016/s0166-2236(02)02191-4 12079762

[B38] Iversen J. R. Balasubramaniam R. (2016). Synchronization and temporal processing. Curr. Opin. Behav. Sci. 8, 175–180. 10.1016/j.cobeha.2016.02.027

[B39] Jauk E. Benedek M. Neubauer A. C. (2012). Tackling creativity at its roots: evidence for different patterns of EEG α activity related to convergent and divergent modes of task processing. Int. J. Psychophysiol. 84, 219–225. 10.1016/j.ijpsycho.2012.02.012 22390860 PMC3343259

[B40] Jung-Beeman M. Bowden E. M. Haberman J. Frymiare J. L. Arambel-Liu S. Greenblatt R.. (2004). Neural activity when people solve verbal problems with insight. PLoS Biol. 2:E97. 10.1371/journal.pbio.0020097 15094802 PMC387268

[B41] Jurcak V. Tsuzuki D. Dan I. (2007). 10/20, 10/10, and 10/5 systems revisited: their validity as relative head-surface-based positioning systems. Neuroimage 34, 1600–1611. 10.1016/j.neuroimage.2006.09.024 17207640

[B42] Kropotov J. D. (2016). Functional Neuromarkers for Psychiatry: Applications for Diagnosis and Treatment. Amsterdam: Academic Press.

[B43] Landau A. T. Limb C. J. (2017). The neuroscience of improvisation. Music Educ. J. 103, 27–33. 10.1177/0027432116687373

[B44] Li C. W. Chen J. H. Tsai C. G. (2015). Listening to music in a risk reward context: the roles of the temporoparital junction and the orbitofrontal/insular cortices in reward-anticipation, reward-gain, and reward-loss. Brain Res. 10, 160–170. 10.1016/j.brainres.2015.10.024 26499261

[B45] Limb C. J. Braun A. R. (2008). Neural substrates of spontaneous musical performance: an fMRI study of jazz improvisation. PLoS One 3:e1679. 10.1371/journal.pone.0001679 18301756 PMC2244806

[B46] Liu S. Chow H. M. Xu Y. Erkkinen M. G. Swett K. E. Eagle M. W.. (2012). Neural correlates of lyrical improvisation: an fMRI study of freestyle rap. Sci. Rep. 2:834. 10.1038/srep00834 23155479 PMC3498928

[B47] Lopata J. A. Nowicki E. A. Joanisse M. F. (2017). Creativity as a distinct trainable mental state: an EEG study of musical improvisation. Neuropsychologia 99, 246–258. 10.1016/j.neuropsychologia.2017.03.020 28322906

[B48] Lu J. Yang H. He H. Jeon S. Hou C. Evans A. C.. (2017). The multiple-demand system in the novelty of musical improvisation: evidence from an MRI study on composers. Front. Neurosci. 11:695. 10.3389/fnins.2017.00695 29311776 PMC5732236

[B49] McPherson M. J. Barrett F. S. Lopez-Gonzalez M. Jiradejvong P. Limb C. J. (2016). Emotional intent modulates the neural substrates of creativity: an fMRI study of emotionally targeted improvisation in jazz musicians. Sci. Rep. 6:18460. 10.1038/srep18460 26725925 PMC4698722

[B50] Mölle M. Marshall L. Wolf B. Fehm H. L. Born J. (1999). EEG complexity and performance measures of creative thinking. Psychophysiology 36, 95–104. 10.1017/s0048577299961619 10098384

[B51] Mullen T. Kothe C. Chi Y. M. Ojeda A. Kerth T. Makeig S. (2013). “Real-time modeling and 3D visualization of source dynamics and connectivity using wearable EEG,” in Proceedings of the 35th Annual International Conference of the IEEE Engineering in Medicine and Biology Society (EMBC) (Osaka, Japan: IEEE), 2184–2187. 10.1109/EMBC.2013.6609968 PMC4119601 24110155

[B52] Müller V. Sänger J. Lindenberger U. (2013). Intra- and inter-brain synchronization during musical improvisation on the guitar. PLoS One 8:e73852. 10.1371/journal.pone.0073852 24040094 PMC3769391

[B53] Newson J. J. Thiagarajan T. C. (2019). EEG frequency bands in psychiatric disorders: a review of resting state studies. Front. Hum. Neurosci. 12:521. 10.3389/fnhum.2018.00521 30687041 PMC6333694

[B54] Nichols T. E. Holmes A. P. (2001). Nonparametric permutation tests for functional neuroimaging: a primer with examples. Hum. Brain Mapp. 15, 1–25. 10.1002/hbm.1058 11747097 PMC6871862

[B55] Pascual-Marqui R. D. (2002). Standardized low-resolution brain electromagnetic tomography (sloreta): technical details. Methods Find. Exp. Clin. Pharmacol. 24, 5–12. 12575463

[B56] Pecenka N. Engel A. Keller P. E. (2013). Neural correlates of auditory temporal predicions during sensorimotor synchronization. Front. Hum. Neurosci. 7:380. 10.3389/fnhum.2013.00380 23970857 PMC3748321

[B57] Pedroni A. Bahreini A. Langer N. (2019). Automagic: standardized preprocessing of big EEG data. Neuroimage 200, 460–473. 10.1016/j.neuroimage.2019.06.046 31233907

[B58] Pinho A. L. deManzano Ö. Fransson P. Eriksson H. Ullén F. (2014). Connecting to create: expertise in musical improvisation is associated with increased functional connectivity between premotor and prefrontal areas. J. Neurosci. 34, 6156–6163. 10.1523/jneurosci.4769-13.2014 24790186 PMC4004805

[B59] Pion-Tonachini L. Kreutz-Delgado K. Makeig S. (2019). ICLabel: an automated electroencephalographic independent component classifier, dataset and website. Neuroimage 198, 181–197. 10.1016/j.neuroimage.2019.05.026 31103785 PMC6592775

[B60] Rangaswamy M. Porjesz B. Chorlian D. B. Wang K. Jones K. A. Bauer L. O.. (2002). β power in the EEG of alcoholics. Biol. Psychiatry 52, 831–842. 10.1016/s0006-3223(02)01362-8 12372655

[B61] Rosen D. S. Erickson B. Kim Y. E. Mirman D. Hamilton R. H. Kounios J. (2016). Anodal tDCS to right dorsolateral prefrontal cortex facilitates performance for novice jazz improvisers but hinders experts. Front. Hum. Neurosci. 10:579. 10.3389/fnhum.2016.00579 27899889 PMC5110534

[B62] Sanyal S. Banerjee A. Mukherjee S. Guhathakurata T. Sengupta R. Ghosh D. (2016). Musical improvisation and brain correlates: an EEG based neurocognitive study using Hindustani music. J. Biomusic. Eng. 4:119 10.4172/2090-2719.1000119

[B63] Schwab D. Benedek M. Papousek I. Weiss E. M. Fink A. (2014). The time-course of EEG α power changes in creative ideation. Front. Hum. Neurosci. 8:310. 10.3389/fnhum.2014.00310 24860485 PMC4026701

[B750] Selim S. Tantawi M. Shedeed H. Badr A. (2018). A CSP\AM-BA-SVM approach for motor imagery BCI system. IEEE Access 6, 49192–49208. 10.1109/ACCESS.2018.286817826737196

[B64] Slobounov S. Cao C. Jaiswal N. Newell K. (2009). Neural basis of postural instability identified by VTC and EEG. Exp. Brain Res. 199, 1–16. 10.1007/s00221-009-1956-5 19655130 PMC2942764

[B65] Sowden P. T. Pringle A. Gabora L. (2015). The shifting sands of creative thinking: connections to dual-process theory. Think. Reason. 21, 40–60. 10.1080/13546783.2014.885464

[B66] Sperandei S. (2014). Understanding logistic regression analysis. Biochem. Med. 24, 12–18. 10.11613/BM.2014.003 24627710 PMC3936971

[B67] Stevens C. E. Zabelina D. L. (2019). Creativity comes in waves: an EEG-focused exploration of the creative brain. Curr. Opin. Behav. Sci. 27, 154–162. 10.1016/j.cobeha.2019.02.003

[B70] Tomioka R. Aihara K. (2007). “Classifying matrices with a spectral regularization,” in ICML ’07: Proceedings of the 24th International Conference on Machine Learning (Corvalis, USA: ACM Press), 895–902.

[B69] Tomioka R. Aihara K. Muller K.-R. (2007). “Logistic regression for single trial EEG classification,” in Advances in Neural Information Processing Systems 19, eds Scholkopf B. Platt J. Hoffman T. (Cambridge, MA: MIT Press), 1377–1384.

[B71] Ventouras E. Ktonas P. Tsekou H. Paparrigopoulos T. Kalatzis I. Soldatos C. (2010). Independent component analysis for source localization of EEG sleep spindle components. Comput. Intell. Neurosci. 2010:329436. 10.1155/2010/329436 20369057 PMC2847376

[B72] Wagner J. Martinez-Cancino R. Makeig S. (2019). Trial-by-trial source-resolved EEG responses to gait task challenges predict subsequent step adaptation. Neuroimage 199, 691–703. 10.1016/j.neuroimage.2019.06.018 31181332 PMC6688934

[B73] Wan X. Crüts B. Jensen H. J. (2014). The causal inference of cortical neural networks during music improvisations. PLoS One 9:e112776. 10.1371/journal.pone.0112776 25489852 PMC4260787

[B74] Warren J. E. Wise R. J. Warren J. D. (2005). Sounds do-able: auditory- motor transformations and the posterior temporal plane. Trends Neurosci. 28, 636–643. 10.1016/j.tins.2005.09.010 16216346

[B75] Winkler I. Debener S. Muller K. R. Tangerm M. (2015). On the influence of high-pass filtering on ICA-based artifact reduction in EEG-ERP. Conf. Proc. IEEE Eng. Med. Biol. Soc. 2015, 4101–4105. 10.1109/embc.2015.7319296 26737196

[B76] Witt S. T. Laird A. R. Meyerand M. E. (2008). Functional neuroimaging correlates of finger-tapping task variations: an ALE meta-analysis. Neuroimage 42, 343–356. 10.1016/j.neuroimage.2008.04.025 18511305 PMC2592684

[B77] Zatorre R. J. Halpern A. R. Bouffard M. (2010). Mental reversal of imagined melodies: a role for the posterior parietal cortex. J. Cogn. Neurosci. 22, 775–789. 10.1162/jocn.2009.21239 19366283

