# Supplementary figures and images for: Music Improvisation Is Characterized by Increase EEG Spectral Power in Prefrontal and Perceptual Motor Cortical Sources and Can be Reliably Classified From Non-improvisatory Performance
Source: Front Hum Neurosci. 2019 Dec 10;13:435. doi: 10.3389/fnhum.2019.00435 (PMC6915035; doi:10.3389/fnhum.2019.00435)

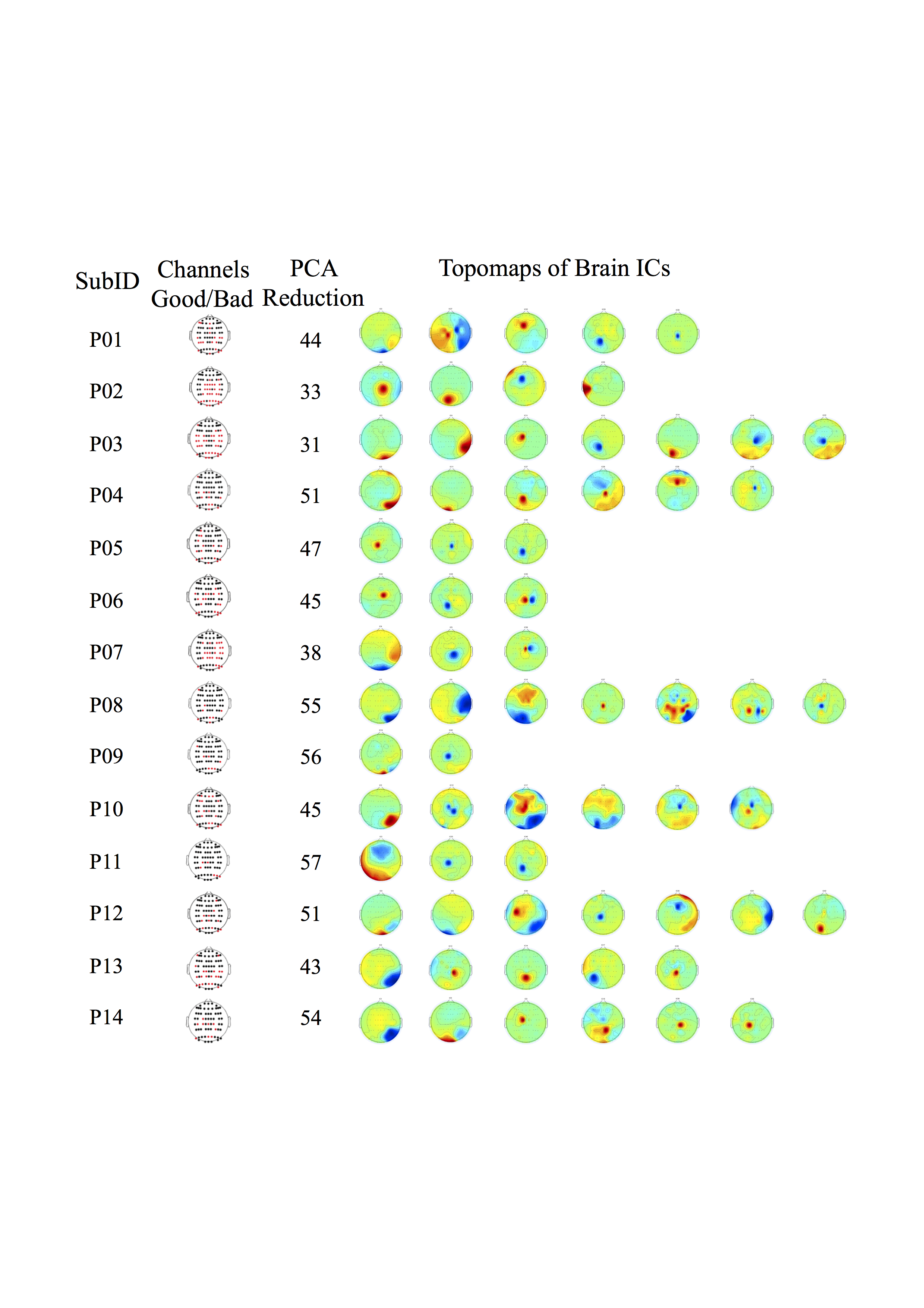

Supplement: FIGURE S1 — Channel and IC information for each participant. The good channels, Black (included in the analysis), and bad channels, Red (removed from analysis), are shown in a topographic plot. The PCA reduction is the number of ICs to retain for ICA. The number is one less than the number of good channels to account for the loss of rank from the average referencing procedure. Topo maps of brain-related independent components ICs. The topographic mapping (topo map) of the scalp data field in a 2-D circular view (nose is at the top) for the brain related ICs determined by ICLabel are given for each of the 14 participants P01 to P14. [file Image_1.TIFF]

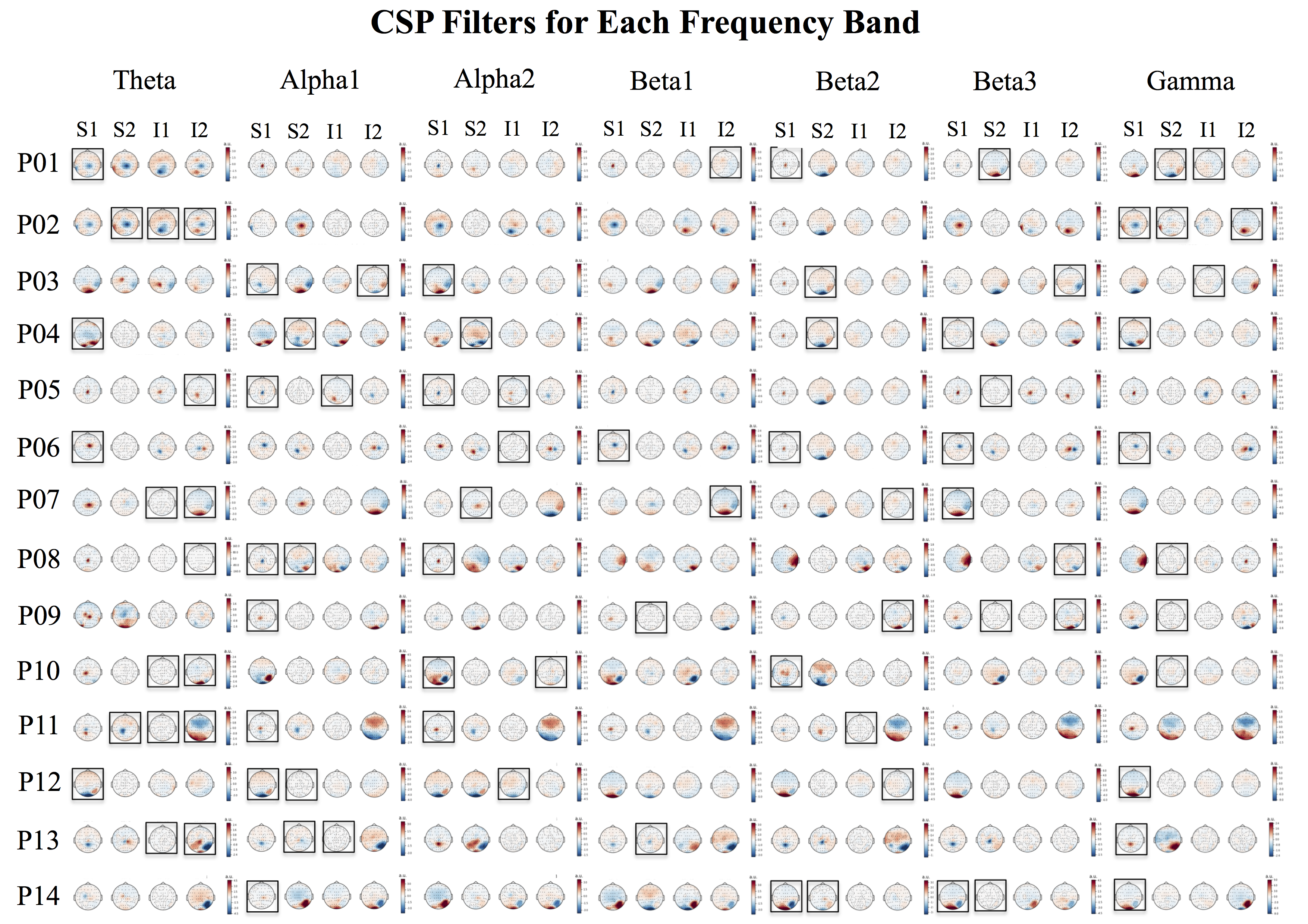

Supplement: FIGURE S2 — Topo maps of the weights of the common spatial pattern (CSP) filters used as features for machine learning. The features for each of the 14 participants are shown consisting of four CSP filters (two for Scale and two for Improv) for each frequency bands (Theta 6.5–8 Hz; Alpha1 8.5–10 Hz; Alpha2 10.5–12 Hz; Beta1 12.5–18 Hz; Beta2 18.5–21 Hz; Beta3 21.5–30 Hz; Gamma 30.5–50 Hz). The weights of the logistic regression machine learning classifier with the top six absolute values of the features are depicted by a black square around the topo map of the CSP filter. [file Image_2.TIFF]

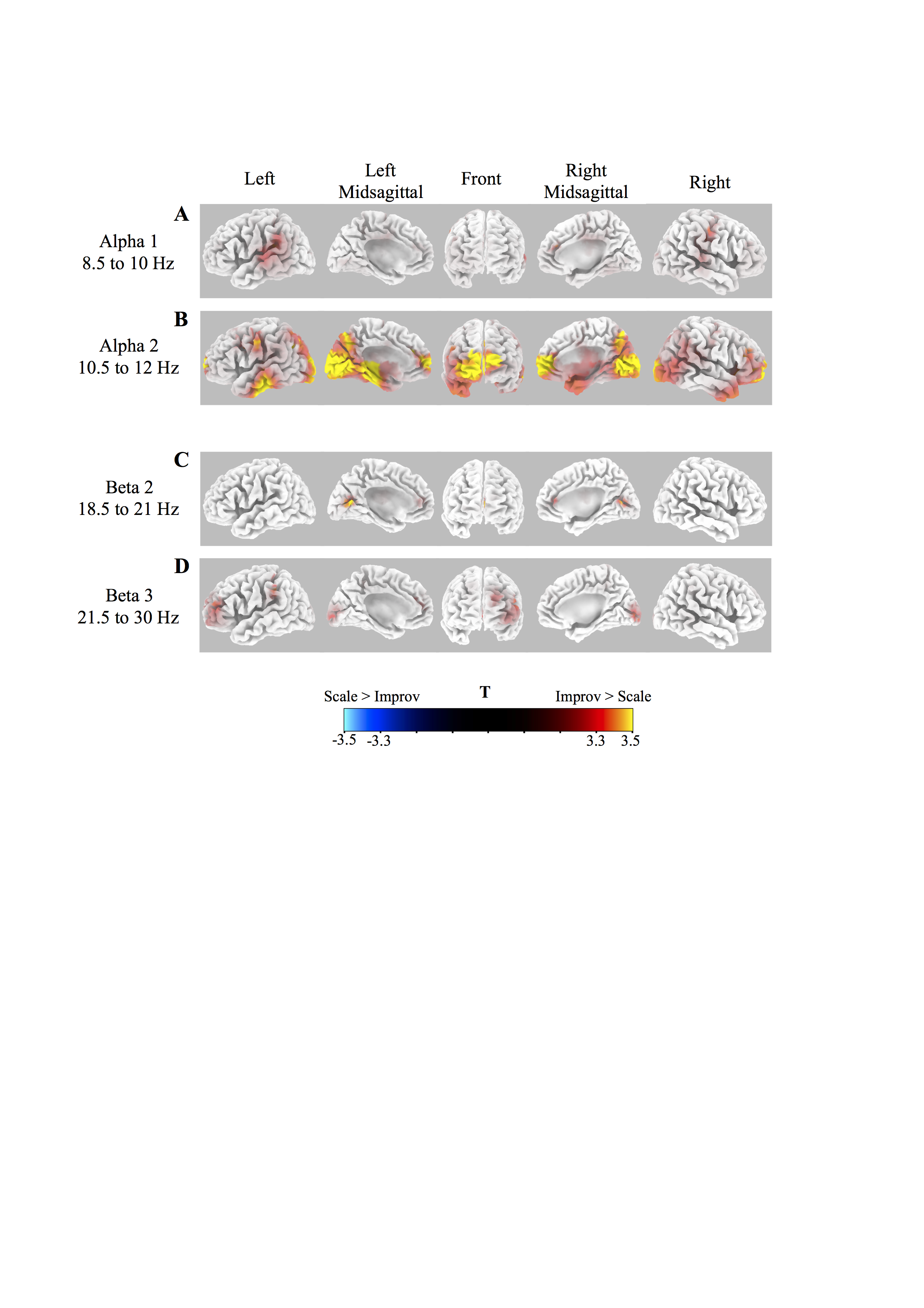

Supplement: FIGURE S3 — Cortical spectral power differences in alpha and beta frequency bands for Improv > Scale with three participants with many interpolated channels removed. Source localization was conducted by sLORETA in the following frequency bands: (A) Alpha1 8.5–10 Hz; (B) Alpha2 10.5–12 Hz; (C) Beta2 18.5–21 Hz; (D) Beta3 21.5–30 Hz. Statistically significantly differential power (Threshold for p < 0.05 correcting for multiple comparisons separately in alpha (SnPM = T > 3.372 two-tailed) and beta (SnPM = T > 3.229 two-tailed) for improv > scale is shown rendered on the surface of the cortex from yellow (high) to red (above threshold). The results are similar to those reported in Figures 2B,C,E,F suggesting to some degree that the sLORETA results are not due to interpolation error. [file Image_3.TIFF]
